# Development and validation of peritumoral vascular and intratumoral radiomics to predict pathologic complete responses to neoadjuvant chemotherapy in patients with triple-negative breast cancer

**DOI:** 10.1186/s12880-024-01311-7

**Published:** 2024-06-06

**Authors:** Tianwen Xie, Jing Gong, Qiufeng Zhao, Chengyue Wu, Siyu Wu, Weijun Peng, Yajia Gu

**Affiliations:** 1 Department of Radiology, Fudan University Shanghai Cancer Center, Shanghai, China; 2 Department of Oncology, Shanghai Medical College, Fudan University, Shanghai, China; 3 Department of Radiology, Longhua Hospital, Shanghai University of Traditional Chinese Medicine, Shanghai, China; 4 Oden Institute for Computational Engineering and Sciences, University of Texas at Austin, Austin, USA; 5 Department of Breast Surgery, Fudan University Shanghai Cancer Center, Shanghai, China

**Keywords:** Triple negative breast neoplasms, Magnetic resonance imaging, Peritumoral vessels, Magnetic resonance angiography, Neoadjuvant therapy

## Abstract

**Background:**

To develop and validate a peritumoral vascular and intratumoral radiomics model to improve pretreatment predictions for pathologic complete responses (pCRs) to neoadjuvant chemoradiotherapy (NAC) in patients with triple-negative breast cancer (TNBC).

**Methods:**

A total of 282 TNBC patients (93 in the primary cohort, 113 in the validation cohort, and 76 in The Cancer Imaging Archive [TCIA] cohort) were retrospectively included. The peritumoral vasculature on the maximum intensity projection (MIP) from pretreatment DCE-MRI was segmented by a Hessian matrix-based filter and then edited by a radiologist. Radiomics features were extracted from the tumor and peritumoral vasculature of the MIP images. The LASSO method was used for feature selection, and the k-nearest neighbor (k-NN) classifier was trained and validated to build a predictive model. The diagnostic performance was assessed using the ROC analysis.

**Results:**

One hundred of the 282 patient (35.5%) with TNBC achieved pCRs after NAC. In predicting pCRs, the combined peritumoral vascular and intratumoral model (fusion model) yields a maximum AUC of 0.82 (95% confidence interval [CI]: 0.75, 0.88) in the primary cohort, a maximum AUC of 0.67 (95% CI: 0.57, 0.76) in the internal validation cohort, and a maximum AUC of 0.65 (95% CI: 0.52, 0.78) in TCIA cohort. The fusion model showed improved performance over the intratumoral model and the peritumoral vascular model, but not significantly (*p* > 0.05).

**Conclusion:**

This study suggested that combined peritumoral vascular and intratumoral radiomics model could provide a non-invasive tool to enable prediction of pCR in TNBC patients treated with NAC.

**Supplementary Information:**

The online version contains supplementary material available at 10.1186/s12880-024-01311-7.

## Introduction

Triple-negative breast cancer (TNBC) is characterized by the lack of the estrogen receptor (ER), progesterone receptor (PR), and human epidermal growth factor receptor 2 (HER2). TNBC, which accounts for 12–15% of all mammary tumors, has a worse outcome compared with other breast cancer subtypes [1]. Currently, neoadjuvant chemotherapy (NAC) is the standard method used to prevent systemic relapse in TNBC patients with locally advanced disease. A pathologic complete response (pCR) to NAC is considered a surrogate marker for improved disease-free survival and overall survival [2]. Although TNBC is the most chemotherapy-responsive tumor of all breast cancer subtypes, there is a high risk of recurrence and high rates of visceral and central nervous metastases in TNBC patients not achieving pCR [3]. To avoid the toxicity of ineffective treatments, it is essential to stratify patients into appropriate treatment groups before the early treatment stages.

Dynamic contrast-enhanced magnetic resonance imaging (DCE-MRI), which depicts and characterizes morphologic and kinetic profiles of tumors and the disorganized, leaky vasculature, is the preferred imaging modality in the NAC setting [4]. Furthermore, radiomics analysis involving computer-based extraction of a large number of quantitative features from DCE-MRI has been shown to improve pCR prediction [5]. Most previous radiomics studies have focused on extracting features from tumor [6–8]. Mazurowski et al. [9] and Wang et al. [10] that evaluated features extracted from tumor-associated background parenchyma enhancement (BPE) in the context of NAC for breast cancer showed an association between this peritumoral radiomics and pCR. In addition, one published study showed that the radiomic descriptor of intratumoral and peritumoral regions on pretreatment DCE-MRI were associated with treatment responses in breast cancer [11]. These evidences indicate that valuable outcome-related information can be found outside of the tumor tissue. Angiogenesis, the biological process in which new blood vessels grow from pre-existing vasculature to provide oxygen and nutrients to tumors, plays a pivotal role in tumor responses to chemotherapy [12]. The exceptionally variable vasculature (in size, shape, and architecture) generates heterogeneous blood flow and limited perfusion throughout the tumor and is essential for cancer proliferation and likely, treatment responses. Therefore, peritumoral vascular and intratumoral features may potentially predict pCR in breast cancer.

In the present study, we aimed to develop and validate a peritumoral vascular and intratumoral radiomics model from pretreatment DCE-MRI to predict pCR in patients with TNBC undergoing surgery after NAC.

## Materials and methods

### Patients

**Fig. 1:**
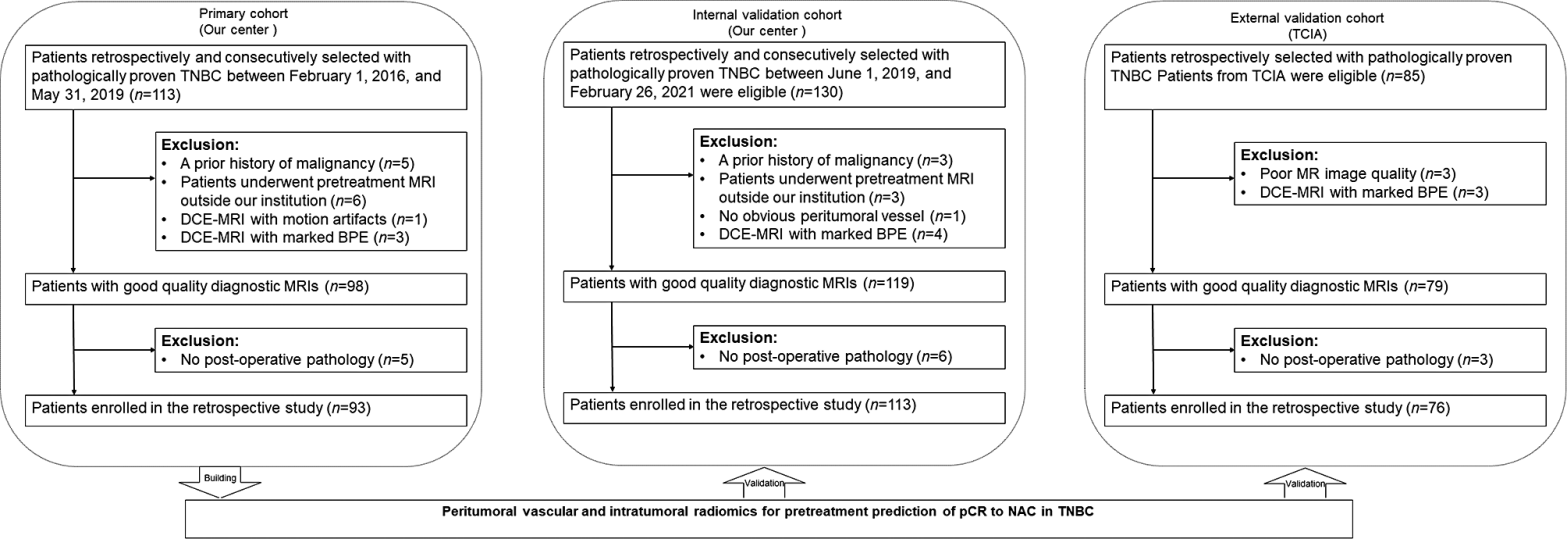
Flowchart of the study population

The retrospective study was approved by the institutional review board of Fudan University Shanghai Cancer Center, and the need to obtain informed consent was waived. In this multicohort study, radiomics analysis was applied to three independent cohorts. A total of 328 women patients diagnosed with breast cancer histologically and TNBC immunohistochemically, and who received complete NAC with no prior treatments, underwent breast MRI before the start of NAC, and underwent surgery after NAC, were included in this study. The exclusion criteria included the following: (*a*) patients with a prior history of malignance (*n* = 8), (*b*) patients without pretreatment MRI or post-operative pathology (*n* = 23), (*c*) patients with poor qualities or motion artifacts on DCE-MRI (*n* = 4), (*d*) patients with marked BPE on DCE-MRI (*n* = 10), (*e*) and patients without obvious peritumoral vessel on DCE-MRI (*n* = 1) (Fig. 1). Finally, the dataset from our center between February 1, 2016 and May 31, 2019 was used as the primary cohort and consisted of 93 patients (mean age, 49 years; range 26–75 years). The dataset from our center between June 1, 2019 and February 26, 2021 was used as the internal validation cohort and consisted of 113 patients (mean age, 47 years; range 25–72 years). The other dataset from “Duke-Breast-Cancer-MRI” of The Cancer Imaging Archive (TCIA) [13] was used as the external validation cohort and consisted of 76 patients (mean age, 49 years; range 24–73 years).

In the primary and internal validation cohorts, ER, PR, HER2, Ki-67 index expression patterns, and axillary lymph node metastatic assessments were obtained from histopathologic reports of core biopsies performed before NAC administration. The immunohistochemical assessment of ER, PR, and HER2 was performed using the standard methods as previously reported [14]. In those tumors that were classified as 2+, HER2 genetic testing was confirmed by fluorescence in situ hybridization.

### Neoadjuvant chemotherapy regimen and response assessment

In the primary and internal validation cohorts, the chemotherapy regimens included epirubicin/cyclophosphamide followed by docetaxel (EC followed by T), docetaxel/carboplatin (TCb), and EC. The median number of NAC cycles was six (range, 4–8). The mean interval between the end of NAC and surgery was 10 days (range, 3–27 days). There were no details of NAC regimens in TCIA cohort. pCR was determined by microscopic examination of the excised tumor and lymph nodes after the completion of NAC and defined as no invasive or noninvasive residual in breast or axillary nodes (*ypT0 ypN0*) [15].

### MRI protocols

The detailed parameters of DCE-MRI acquisition of all cohorts can be found in Appendix E1 in the Supplementary Material. In the primary and internal validation cohorts, all breast MR examinations were performed within 14 days before the start of NAC. DCE-MRI was performed using a fat-suppressed T1-weighted 3D fast spoiled gradient-echo sequence before and five times continuously. The first postcontrast phase was performed twenty seconds after a bolus injection of a gadolinium contrast agent (Magnevist, Bayer HealthCare Pharmaceuticals Inc.). The injections were performed with an automatic injector (OptiStar® Elite, Liebel-Flarsheim) at a dose of 0.1 mmol per kilogram of body weight and rate of 2 ml/sec, followed by a 20 mL saline flush. The subtraction and axial MIP images were generated automatically after acquisition.

In TCIA dataset, the contrast agents included Gadavist, Magnevist, and Multihanc with the volume of 10–20 ml. The subtraction and axial MIP images were manually calculated by the radiologist (TX, 5 years of experience).

### Tumor segmentation and peritumoral vessel segmentation

All MR images were reviewed by two breast radiologists (TX, 5 years of experience; and QZ, 11 years of experience), who were blinded to the results of the treatment outcomes. For patients with multifocal or multicentric tumors, the tumors with the largest size and the ipsilateral vessel were segmented and analyzed on the basis of the axial MIP of the first postcontrast phase.

Tumor segmentation on the MIP image was conducted manually by the breast radiologist (TX, 5 years of experience). The region of interest (ROI) was delineated to include the entire tumor by using a free open-source software package (ITK-SNAP, version 3.8.0; http://itk-snap.org). If there was overlap between the index tumor and the peritumoral vessel in the axial MIP image, the intersection was removed using the eraser tool. The illustration for tumor segmentation can be found in Appendix E2 in the Supplementary Material.

**Fig. 2:**
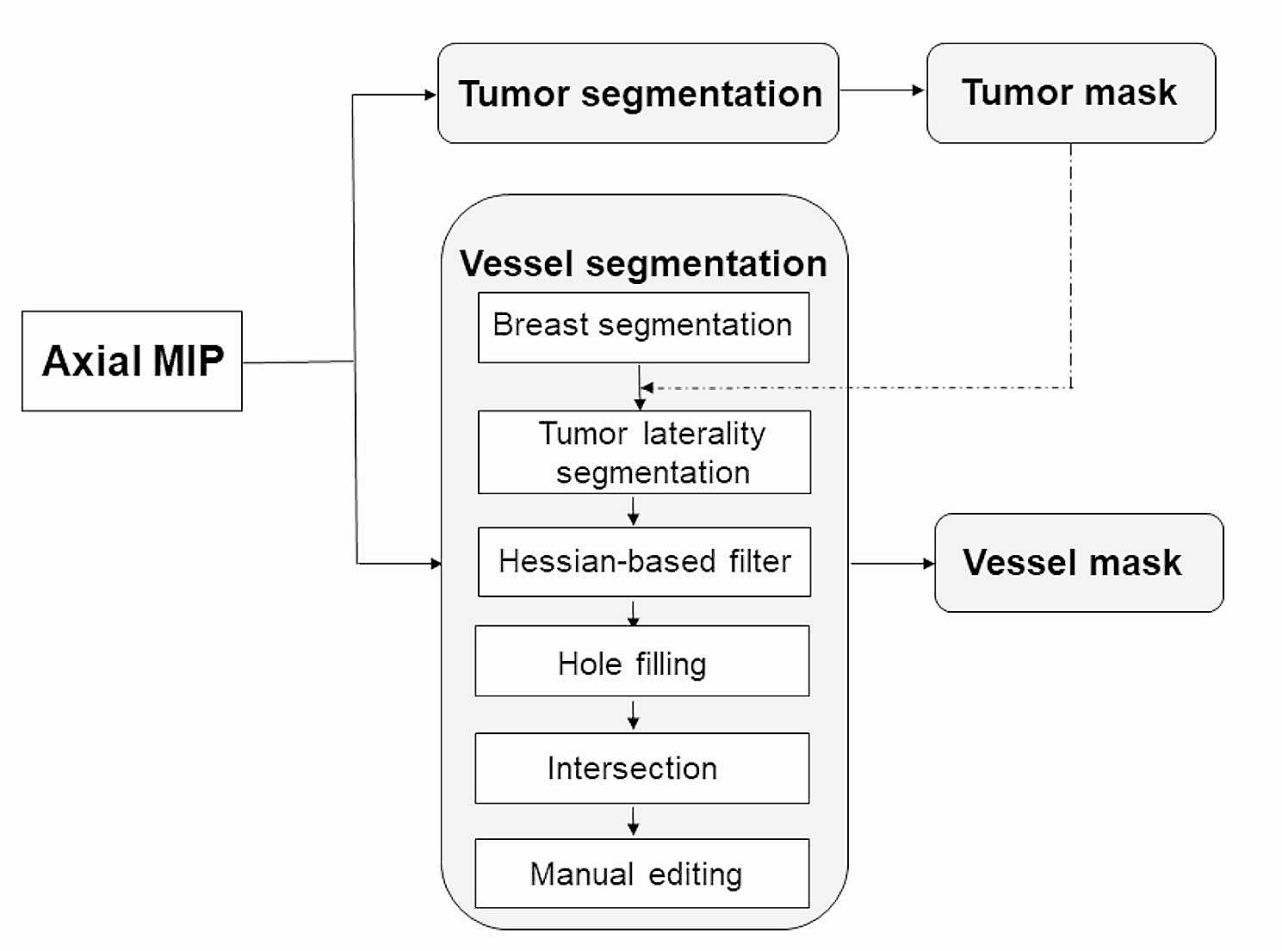
Flowchart of the tumor and peritumoral vessel segmentation procedure. Tumor and peritumoral vessel segmentations were performed on the axial maximum intensity projection (MIP) of the first postcontrast phase. After breast segmentation, the lateral breast index tumor was segmented according to the tumor location. Peritumoral vessel on the MIP image was segmented using a multiscale Hessian-based filter. Additionally, the peritumoral vasculature by algorithm segmentation was generated via the intersection of the lateral tumor breast mask and the binary vessel segmentation region after reducing small gaps and filling holes. Finally, the vessel mask was identified via manual editing

**Fig. 3:**
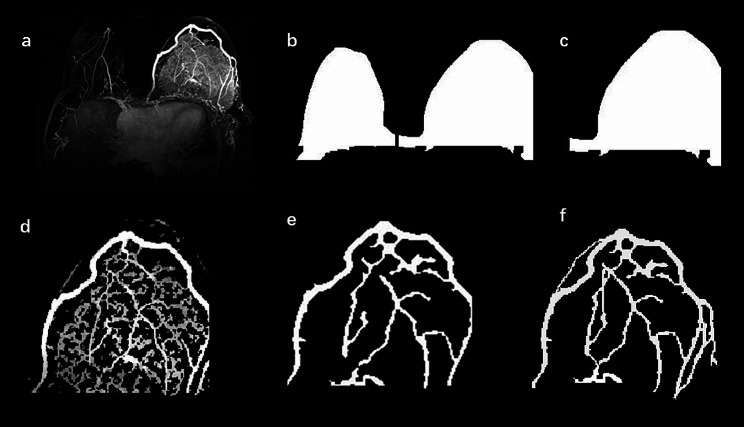
An illusion of vessel segmentation. (**a**) The axial maximum intensity projection (MIP) image in one patient. After the anatomic breast segmentation was performed (**b**), the tumor laterality was segmented according to the tumor location (**c**). Peritumoral vessel in the MIP image were enhanced and segmented with a multiscale Hessian-based filter (**d**). After hole filling and intersection steps were performed, peritumoral vasculature by algorithm segmentation was identified (**e**). The final vessel mask was identified via manual editing (**f**)

The enhancement and segmentation of peritumoral vessel were performed by the eigenvalue analysis of the multiscale Hessian-based filter, which showed simultaneous noise and background suppression and vessel enhancement in MIP images [16]. The details of the multiscale Hessian-based filter method and peritumoral vessel segmentation by algorithm can be found in Appendix E3 and E4 in the Supplementary Material. The segmentation of peritumoral vessel were performed with the Python programming language (Scikit-image package, v. 3.6, Python Software Foundation, https://www.python.org/). Then, the peritumoral vasculature by algorithm segmentation was loaded to ITK-SNAP again, and a senior breast radiologist (QZ, 11 years of experience) performed manual editing by painting missing voxels and erasing incorrect voxels to get the final peritumoral vasculature. The manual vessel editing procedure took approximately 4 min per case. The flowchart and illustration for the vessel segmentation procedures are shown in Figs. 2 and 3.

The final peritumoral vasculature, checked and edited by the breast radiologist (QZ, 11 years of experience), represented the reference standard. To evaluate the performance of vessel detection by algorithm segmentation, the correct-detection rate, incorrect-detection rate, and missed-detection rate were computed (Appendix E5 in the Supplementary Material).

### Radiomic feature extraction

After tumor and peritumoral vessel were segmented, the shape, statistical and textural features were extracted on MIP images using the PyRadiomics Python package [17]. For the tumor and peritumoral vessel detected on the MIP images, we extracted radiomics features, including 10 shape features, 19 first-order statistical features, and 70 texture features. Furthermore, we extracted 356 wavelet features (i.e., LL, LH, HL, HH) for each tumor. Wavelet features provide representative transformed domain information regarding intensity and textural features by decomposing the original image in low and high frequencies [18]. Finally, 455 features quantifying intratumoral characteristics and 99 features quantifying peritumoral vascular characteristics were obtained.

### Radiomic feature selection and model development

All the radiomics features were scaled to a range of [0, 1] by using a minimum-maximum scaler. Then, the least absolute shrinkage and selection operator (LASSO) configured recursive feature elimination (RFE) method was applied to select features for the intratumoral model, peritumoral vascular model, individually. And only the training dataset was used to train the LASSO feature selector. The k-nearest neighbor (k-NN) classifier was used to train and test the radiomics models for predicting pCR to NAC. The k-NN (k = 5) technique was trained based on the primary cohort, and then tested in the internal and external validation cohorts.

After building the tumor features-based prediction model and vessel features-based prediction model, an information fusion method was applied to fuse the prediction scores generated by the two models to improve the model performance [19]. The information fusion method included the minimum, maximum, and weighting average of the fusion.

### Statistical analysis

Comparisons between the patient groups were employed with the Chi-square test or Fisher’s test for qualitative variables and the Student’s *t*-test or Mann-Whitney *U* test for quantitative variables. The areas under the receiver operating characteristic (ROC) curves (AUCs) were assessed and compared among the intratumoral model, peritumoral vascular model, and fusion model using the DeLong method [20]. Statistical analyses and radiomics analyses were performed with the Python programming language (v. 3.6, Python Software Foundation, https://www.python.org/). In our implementation, we utilized the default parameters provided by the Python packages for building the machine-learning classifier. Regarding the min-max normalization used in our method, it was applied consistently to maintain the scale of variables across the dataset. *p*$<$0.05 was considered statistically significant.

## Results

### Patients and pathologic complete responses

In total, 282 patients with TNBC were finally enrolled in this study. The clinical pathologic characteristics of patients from all cohorts are listed in Table 1.

**Table 1:** The clinicopathologic characteristics of the patients with TNBC in the three cohorts

Characteristic	Primary cohort (*n* = 93)			Internal validation cohort (*n* = 113)					External validation cohort (*n* = 76)		
	pCR (*n* = 34)	Non-pCR (*n* = 59)	***p***	pCR (*n* = 44)	Non-pCR (*n* = 69)		***p***		pCR (*n* = 22)	Non-pCR (*n* = 54)	***p***
Age, mean ± SD, years	46.59 ± 11.42	50.90 ± 11.14	0.078	45.32 ± 9.37	48.13 ± 11.85		0.186		47.77 ± 14.17	49.83 ± 10.49	0.487
Menopausal status			0.012^*^				0.434				0.723
Peri or Postmenopausal	11	35		19	35				8	22	
Premenopausal	23	24		25	34				14	32	
Clinical stage			0.946				0.042^*^				0.819
I/II	25	43		32	37				17	43	
III/IV	9	16		12	32				5	11	
Lesion size, mean ± SD, mm	33.74 ± 15.95	43.25 ± 20.59	0.022^*^	36.09 ± 19.34	46.72 ± 26.68		0.024^*^		27.91 ± 16.94	34.30 ± 21.48	0.217
Axillary LN before NAC			0.679				0.327				0.191
Negative	9	18		11	12				13	23	
Positive	25	41		33	57				9	31	
Ki-67 before NAC			0.121				0.480				NA
Negative	0	4		2	6				NA	NA	
Positive	34	55		42	63				NA	NA	
Enhancement Pattern			0.083				0.018^*^				0.162
With nonmass enhancement	4	16		4	19				1	11	
Mass only	30	43		40	50				21	43	
Rim Enhancement			0.640				0.066				0.468
Negative	19	30		21	45				15	32	
Positive	15	29		23	24				7	22	
Chemotherapy regimen			0.616				0.430				NA
EC-T	25	42		24	29				NA	NA	
EC	2	7		5	10				NA	NA	
TCb	7	10		15	30				NA	NA	

*EC* epirubicin with cyclophosphamide; *EC-T* epirubicin with cyclophosphamide plus docetaxel; *LN* lymph node; *NAC* neoadjuvant chemotherapy; *pCR* pathologic complete response; *SD* standard deviation; *TCb* docetaxel with carboplatin.

^*^p < 0.05.

A hundred of 282 patients (35.5%) achieved pCRs after NAC. The pCR rates in the primary cohort, internal validation cohort, and TCIA cohort were 36.6%, 38.9%, and 29.0%, respectively. With regard to clinicopathologic characteristics, no differences between the pCR and non-pCR groups in all cohorts were found in terms of the axillary status, Ki-67 expression, rim enhancement sign, or chemotherapy regimen (*p* > 0.05). Patients who achieved pCR in the primary cohort had greater premenopausal status, and had smaller tumor sizes than those who did not (*p* = 0.012, 0.022, respectively). Meanwhile, pCR was found to be significantly associated with clinical stage, tumor size and enhancement pattern in the internal validation cohort (*p* = 0.042, 0.024, 0.018, respectively).

### Feature extraction

**Fig. 4:**
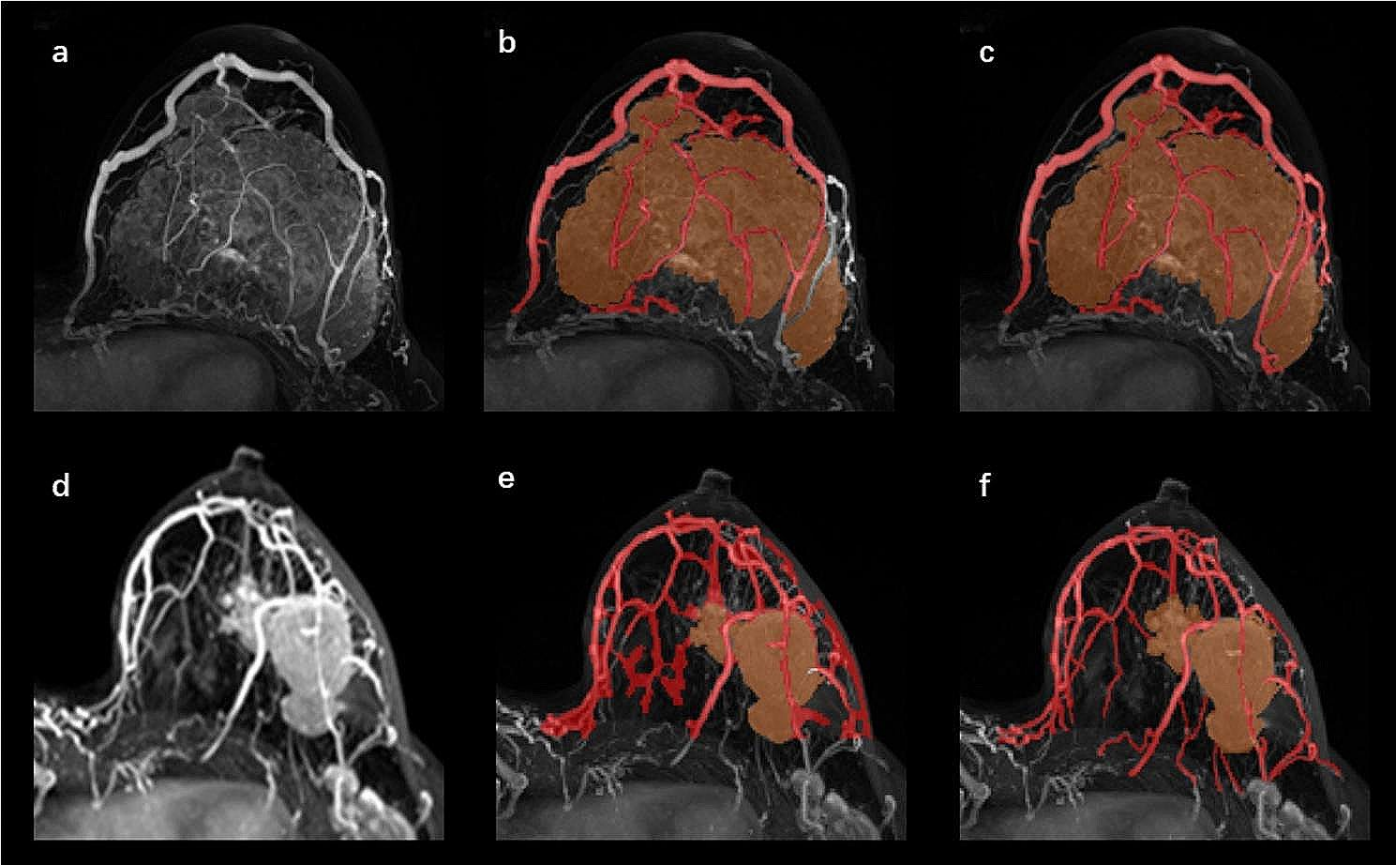
Examples of vessel segmentation in two representative patients are shown. The upper row shows a patient with triple-negative breast cancer (TNBC) who did not achieve a pathologic complete response (pCR); the lower row shows a patient with TNBC who achieved a pCR. (**a**) and (**d**) are maximum intensity projection (MIP) images. (**b**) and (**e**) are peritumoral vessel segmented by algorithm and intratumoral segmentation. (**c**) and (**f**) are peritumoral vessel edited by the radiologist and intratumoral segmentation

The overall performance of vessel identification was evaluated on all cohorts (Table 2). Vessel segmentation examples of 2 representative patients are shown in Fig. 4.

**Table 2:** Vessel detection algorithm performance

	Correct-detection rate (%)	Incorrect-detection rate (%)	Missed-detection rate (%)
Primary cohort			
Range	60–100	0–45	0–40
Median	90.0	20.9	10.0
Mean (SD)	89.8 (5.2)	20.1 (6.5)	10.2 (5.2)
Internal validation cohort			
Range	64–100	0-42.9	0–36
Median	86.4	25.6	13.6
Mean (SD)	85.1 (7.1)	24.9 (7.3)	14.9 (7.1)
External validation cohort			
Range	58.3–100	0–40.0	0-41.7
Median	84.3	27.5	15.7
Mean (SD)	83.1 (9.1)	27.2 (8.2)	16.9 (9.1)

*SD* standard deviation.

Eight tumor features and nigh peritumoral vessel features were selected from initial feature pool were included for further analysis. Detailed information on selected features is shown in Table 3.

**Table 3:** Radiomics features extracted from the tumor and peritumoral vessel were identified after feature selection

Location	Feature family	Feature	Description
Tumor (wavelet-LL)	GLDM	High Gray Level Emphasis	Distribution of the higher gray-level values
		Large Dependence Emphasis	Distribution of large dependencies
		Large Dependence High Gray Level Emphasis	Joint distribution of large dependence with higher gray-level values
		Large Dependence Low Gray Level Emphasis	Joint distribution of large dependence with lower gray-level values
		Low Gray Level Emphasis	Distribution of low gray-level values
		Small Dependence Emphasis	Distribution of small dependencies
		Small Dependence High Gray Level Emphasis	Joint distribution of small dependence with higher gray-level values
		Small Dependence Low Gray Level Emphasis	Joint distribution of small dependence with lower gray-level values
Vessel	GLDM	Gray Level Variance	Variance in grey level in the image
		High Gray Level Emphasis	Distribution of the higher gray-level values
		Large Dependence Emphasis	Distribution of large dependencies
		Large Dependence High Gray Level Emphasis	Joint distribution of large dependence with higher gray-level values
		Large Dependence Low Gray Level Emphasis	Joint distribution of large dependence with lower gray-level values
		Low Gray Level Emphasis	Distribution of low gray-level values
		Small Dependence Emphasis	Distribution of small dependencies
		Small Dependence High Gray Level Emphasis	Joint distribution of small dependence with higher gray-level values
		Small Dependence Low Gray Level Emphasis	Joint distribution of small dependence with lower gray-level values

*GLDM* Gray Level Dependence Matrix.

### Performance of radiomics models

The AUCs and ROC curves of the radiomics analyses in all cohorts are shown in Table 4. Other relevant classification metrics including accuracy, precision, recall, sensitivity, and specificity are shown in Appendix E6 in the Supplementary Material.

**Table 4:** Performance of the peritumoral vascular radiomics model and intratumoral radiomics model

	Primary cohort		Internal validation cohort		External validation cohort	
	AUC	95% CI	AUC	95% CI	AUC	95% CI
Tumor^a^	0.75	[0.66, 0.81]	0.64	[0.53, 0.73]	0.61	[0.47, 0.74]
Vessel^b^	0.77	[0.69, 0.83]	0.65	[0.54, 0.73]	0.61	[0.47, 0.73]

Tumor^a^: prediction score generated using the intratumoral features-based model; Vessel^b^: prediction score generated using the peritumoral vascular features-based model.

AUC, area under the curve; CI, confidence interval.

The peritumoral vascular model resulted in an AUC ranging from 0.61 to 0.77: primary cohort, 0.77 (95% confidence interval [CI]: 0.69, 0.83); internal validation cohort, 0.65 (95% CI: 0.54, 0.73); TCIA cohort, 0.61 (95% CI: 0.47, 0.73). Meantime, the intratumoral model yielded an AUC ranging from 0.61 to 0.75: primary cohort, 0.75 (95% CI: 0.66, 0.81); internal validation cohort, 0.64 (95% CI: 0.53, 0.73); TCIA cohort, 0.61 (95% CI: 0.47, 0.74). There were no statistically significant differences in each cohort of AUCs using intratumoral features or peritumoral vascular features (*p* > 0.05).

**Fig. 5:**
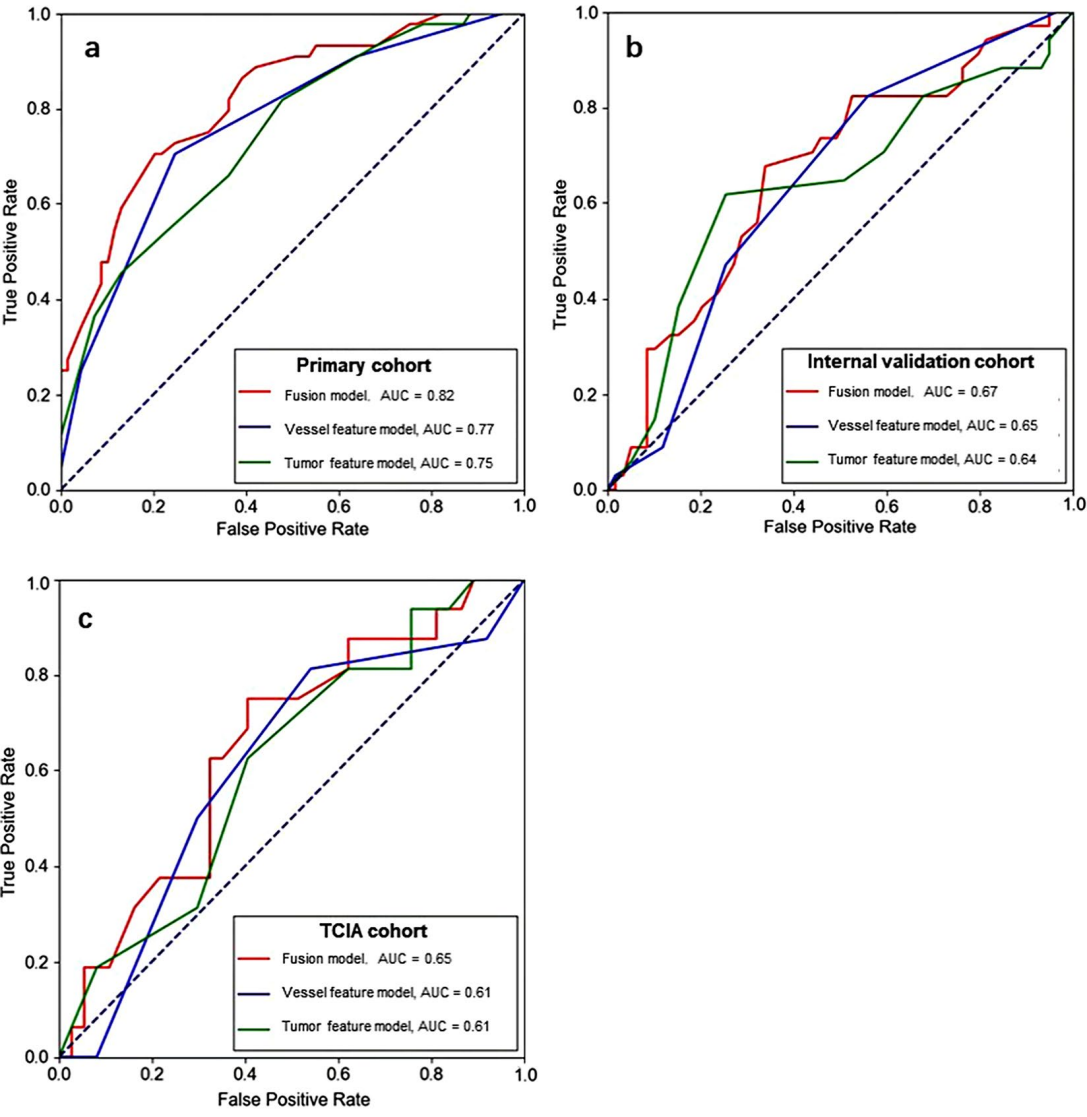
Receiver operating characteristic (ROC) curves generated using the three different radiomics models in the primary (**a**), internal validation (**b**), and TCIA (**c**) cohorts. The models included one that only used peritumoral vessel features (green), one that only used tumor features (blue), and the best fusion model (red)

The fusion model yielded the highest AUC of 0.82 (95% CI: 0.75, 0.88) in the primary cohort, and the highest AUC of 0.67 (95% CI: 0.57, 0.76) in the internal cohort and the highest AUC of 0.65 (95% CI: 0.52, 0.78) in TCIA cohort (Table 5; Fig. 5). The fusion model showed improved performance over the intratumoral model and the peritumoral vascular model, but not significantly (*p* > 0.05).

**Table 5:** A summary of the area under the curve (AUC) values obtained using different fusion methods to combine prediction scores generated by tumor features and peritumoral vessel features

	Primary cohort		Internal validation cohort		External validation cohort	
Model	AUC	95% CI	AUC	95% CI	AUC	95% CI
Minimum	0.81	[0.74, 0.87]	0.65	[0.54, 0.74]	0.63	[0.49, 0.76]
Maximum	0.76	[0.68, 0.83]	0.65	[0.54, 0.74]	0.59	[0.44, 0.70]
0.9×Tumor^a^+0.1×Vessel^b^	0.80	[0.72, 0.86]	0.67	[0.57, 0.76]	0.64	[0.50, 0.77]
0.8×Tumor + 0.2×Vessel	0.80	[0.72, 0.86]	0.67	[0.57, 0.76]	0.64	[0.50, 0.77]
0.7×Tumor + 0.3×Vessel	0.81	[0.73, 0.87]	0.67	[0.56, 0.75]	0.65	[0.51, 0.77]
0.6×Tumor + 0.4×Vessel	0.82	[0.75, 0.88]	0.67	[0.55, 0.75]	0.65	[0.52, 0.78]
0.5×Tumor + 0.5×Vessel	0.82	[0.74, 0.88]	0.67	[0.56, 0.76]	0.64	[0.51, 0.77]
0.4×Tumor + 0.6×Vessel	0.81	[0.73, 0.87]	0.66	[0.55, 0.75]	0.62	[0.48, 0.76]
0.3×Tumor + 0.7×Vessel	0.81	[0.73, 0.87]	0.66	[0.55, 0.75]	0.62	[0.49, 0.76]
0.2×Tumor + 0.8×Vessel	0.78	[0.70, 0.85]	0.65	[0.54, 0.74]	0.62	[0.48, 0.76]
0.1×Tumor + 0.9×Vessel	0.77	[0.68, 0.83]	0.65	[0.54, 0.74]	0.61	[0.46, 0.73]

Tumor^a^: prediction score generated using the intratumoral features-based model; Vessel^b^: prediction score generated using the peritumoral vascular features-based model.

*AUC* area under the curve; *CI* confidence interval.

## Discussion

In this study, we developed and validated a radiomics model that incorporated peritumoral vascular and intratumoral features extracted from pretreatment MIP images to predict pCRs to NAC in patients with TNBC. The proposed radiomics model provides new insights into the biological characteristics of TNBC and the early prediction of its pathologic responses to NAC.

Identifying patients not likely to benefit from NAC before treatment could enable tailored individual patient therapies, especially patients with TNBC, which has been known to display the highest distant metastatic rates and lowest overall survival of all breast cancer subtypes. Previous studies have shown that the prediction of pCRs to NAC varied across biological subtypes, indicating the need for specific radiomics models [3, 15]. A radiomics model dedicated to a specific biological subtype could create more reproducible and robust classification results [21].

Previous studies using intratumoral texture features extracted from DCE-MRI yielded AUCs of 0.64–0.68 for the early prediction of pCR in patients with TNBC [7, 22] and were in accordance with those in our study using texture features extracted from 2D MIP. The all intratumoral texture features selected were obtained from wavelet images, which are high-dimensional features that cannot be perceived by humans but hold more detailed information about tumors and are more sensitive when predicting pCRs [7, 23]. Although MIPs miss the proportion of tumor intensity, MIPs integrated with a DCE-MRI protocol can reveal not only the visualization of enhancing tumor but also the tumor-associated vasculature in the clinical scenario, and simplify the workflow to perform the extraction of tumor and peritumoral vasculature in the only one image. To the best of our knowledge, MIPs have never been proposed for feature extractions. The potential association between MIP-derived tumor features and pCR should be further investigated in future studies.

Tumor angiogenesis is essential for the growth, invasion, and metastasis of tumors. Overexpression of vascular endothelial growth factor (VEGF) has been extensively investigated to be a key player in the formation of tumor neovasculature with many abnormal features [24]. Compared with ER-positive breast cancer, TNBC has a higher degree of VEGF, an avid stimulator of angiogenesis, which is closely correlated with the risk of distant metastases [25]. This angiogenic activity constitutes the basis for the detection and differentiation of breast cancer using DCE-MRI. MIPs from DCE-MRI can assess angiogenic activity and are considered a promising noninvasive investigational tool. Studies focusing on the use of peritumoral vessel to evaluate the response of patients with breast cancer to NAC have been reported [26, 27]. These studies assessed quantitative differences in the number and volume of peritumoral vessels before and after NAC and showed that vessel changes could serve as an early indicator to predict pathologic responses.

In our study, we performed Hessian-based algorithm to segment the tumor-associated vessels from the axial MIPs of the first postcontrast phase where the greatest lesion conspicuity with the lowest background parenchymal enhancement were demonstrated, as well as the best “angiographic effect” for both arteries and veins [28]. Hessian-based algorithm showed correct-detection rates of 83.1-89.8%, incorrect-detection rates of 20.1-27.2%, and missed-detection rates of 10.2-16.9%, which are similar to those in previous studies with the same algorithm [29, 30]. The incorrect-detection rates were mainly caused by linearly distributed BPE, as well as subtraction artifacts along the breast skin. The missed-detection rates were mainly due to low-signal vascular pixels identified by the radiologist but not detected by the algorithm. Furthermore, the senior radiologist checked and edited the vasculature segmented by the algorithm to get the final peritumoral vasculature for the further radiomics feature extraction. The best-performing vessel features were all from gray level dependence matrix (GLDM) quantifying gray level dependencies in an image. There features may indicate more heterogeneous of abnormal angiogenic vessels surrounding tumors demonstrating non-pCRs [31]. A higher level of abnormal vasculature and the possibility of more discontinuities in the convoluted vasculature might constrict the delivery of chemotherapeutic drugs to tumors, thereby resulting in worse treatment responses [32].

Peritumoral vascular model of TNBC on pretreatment MIP images demonstrated a similar classification performance to that of intratumoral model. Furthermore, a combined peritumoral vascular and intratumoral signature resulted in improved performance, albeit the difference was not significant. These findings suggest that peritumoral vascular radiomics based on MIP might provide a preliminary success for treatment responses in patients with TNBC. However, the performance of our model in the external validation cohort was observed to be subpar. Factors such as differences in patient demographics and treatment protocols likely contributed to this discrepancy. Moving forward, in future studies, addressing these limitations and refining the model through additional validation steps will be paramount to enhancing its reliability and applicability in real-world clinical settings.

For this study, we acknowledge the following limitations. First, the models in the retrospective study did not used these certain imaging-related features, such as tumor size and enhancement pattern, which exhibited a positive correlation with pCR prediction in one or two cohorts. Further prospective studies will explore these imaging-based features to enhance the performance of the pCR prediction model. Second, we extracted tumor features from a single representative 2D MIP image, which might not provide a comprehensive assessment of whole-tumor heterogeneity. Also, the vasculature segmented from the 2D MIP image could give distorted measures. Specifically, we used the axial MIP image to extract peritumoral vascular and intratumoral features because the MIP image revealed not only tumor enhancements but also tumor vasculature, making the interpretation and analysis simpler than when using full-study DCE-MRI images. We are currently exploring 3D tumor (including necrosis or excluding necrosis) and vascular segmentation on 3D-subtracted postcontrast images [33]. Third, the evaluation of peritumoral vessels was not compared with vessels on the contralateral normal breast in our study. Further studies evaluating vessels of the contralateral normal breast are needed. Finally, MR contrast agent, MR devices, scanning parameters such as spatial resolution of images, and different phases of the menstrual cycle have effects on the segmentation of peritumoral vessels and so on the performance of peritumoral vascular radiomics analysis.

## Conclusions

The peritumoral vascular and intratumoral radiomics based on pretreatment MIP images from DCE-MRI can be used to predict pCR to NAC in TNBC patients. This strategy of radiomics analysis could provide a potential approach to assist in understanding the biologic behavior, pretreatment planning, and response prediction of TNBC.

### Electronic supplementary material

Below is the link to the electronic supplementary material.

Supplementary Material 1

## Data Availability

The datasets used and/or analysed during the current study are available from the corresponding author upon reasonable request.

## Abbreviations

AUC: Areas under the curve
BPE: Background parenchyma ehancement
DCE-MRI: Dynamic contrast-enhanced magnetic resonance imaging
GLDM: Gray level dependence matrix
k-NN: k-nearest neighbor
LASSO: Least absolute shrinkage and selection operator
MIP: Maximum intensity projection
NAC: Neoadjuvant chemotherapy
pCR: Pathologic complete response
RFE: Recursive feature elimination
ROC: Receiver operating characteristic
TNBC: Triple-negative breast cancer
