# Supplementary material for: Development and validation of peritumoral vascular and intratumoral radiomics to predict pathologic complete responses to neoadjuvant chemotherapy in patients with triple-negative breast cancer
Source: BMC Med Imaging. 2024 Jun 6;24:136. doi: 10.1186/s12880-024-01311-7 (PMC11155097; doi:10.1186/s12880-024-01311-7)
Supplement: Supplementary file 1 — Supplementary Material 1 [file 12880_2024_1311_MOESM1_ESM.docx]

**Appendix E1**

**DCE-MRI scanning parameters for the patients.**

| **Dataset** | **Scanner** | **Patients no.** | **TR/TE**  **(ms)** | **Flip Angle** | **FOV**  **(mm^2^)** | **Matrix** | **Slice thickness (mm)** | **Slices** | **Temporal**  **resolution** | **Acquisition**  **Time** |
| --- | --- | --- | --- | --- | --- | --- | --- | --- | --- | --- |
| **Primary cohort**  **(Our center)** | Siemens 3.0T  (Skyra) | 93 | 4.5/1.6 | 10° | 340 | 384×384 | 1.5 | 80 | 1min | 6min |
| **Internal validation cohort**  **(Our center)** | Siemens 3.0T  (Skyra) | 78 | 4.5/1.6 | 10° | 340 | 384×384 | 1.5 | 80 | 1min | 6min |
|  | Siemens 3.0T  (Prisma) | 5 | 3.7/1.5 | 10° | 340 | 384×384 | 1.8 | 96 | 1min2s | 6min12s |
|  | GE 3.0T  (Pioneer) | 20 | 7.6/4.3 | 10° | 336 | 288×288 | 2.2 | 76 | 59s | 5min54s |
|  | GE 1.5T (Creator) | 10 | 6.9/3.3 | 10° | 312 | 256×200 | 2.2 | 68 | 59s | 5min54s |
| **External validation cohort**  **(TCIA)** | Siemens 3.0T  (Skyra) | 12 | 3.8/1.4 | 10° | 350-440 | 448×381 | 1.1-2.0 | 208, 224 | 1min39s-2min3s | 4min57s-6min9s |
|  | Siemens 3.0T  (Trio) | 3 | 3.7-4.1/1.4-1.5 | 10° | 320, 340 | 448×448 | 1.1-1.4 | 128, 160 | 1min49s-2min15s | 5min27s-9min |
|  | GE 3.0T  (HDx) | 35 | 5.1-7.0/2.3-2.7 | 10° | 300-390 | 350×350, 384×384 | 1.1-2.0 | 142-188 | 1min39s-2min22s | 4min57s-9min28s |
|  | Siemens 1.5T  (Avanto) | 15 | 4.1-4.7/1.3-1.5 | 10°, 12° | 300-400 | 320×320, 448×448 | 1.1-2.0 | 160-192 | 1min58s-2min6s | 5min54s-8min24s |
|  | GE 1.5T  (MR450w) | 2 | 5.2-5.4/2.4-2.5 | 10° | 360 | 512×512 | 1.1-2.0 | 174-178 | 1min53s-2min | 5min51s-6min |
|  | GE 1.5T  (HDxt) | 9 | 4.9-5.2/2.2-2.4 | 10° | 320-400 | 340×340 | 1.1-2.0 | 144-200 | 1min28s-1min59s | 3min46s-5min57s |

FOV field of view; TCIA The Cancer Imaging Archive; TR repetition time; TE echo time.

**Appendix E2**


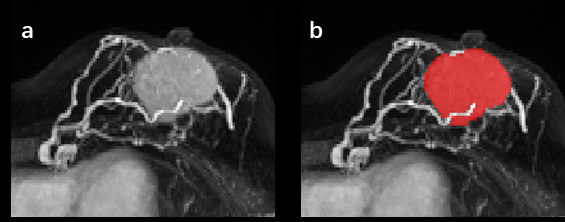


**The illustration for tumor segmentation.** Tumor mask identification (b) on an axial maximum intensity projection (MIP) image (a). The region of interest was delineated as large as possible to include the entire tumor on the axial MIP image. Then an intersection between the index tumor and vessel was removed using the eraser tool.

**Appendix E3**

**The multiscale Hessian-based filter method.** The Hessian matrix provides principal direction and vesselness measurements, which are calculated as the maximum probability that each pixel belongs to a vessel. Given image I, each element in the Hessian matrix gives a second-order directional derivative (Equation 1),

$H=\left( \begin{matrix} \frac{\partial^{2}I}{{\partial x}^{2}} & \frac{\partial^{2}I}{\partial_{x}\partial_{y}} \\ \frac{\partial^{2}I}{\partial_{y}\partial_{x}} & \frac{\partial^{2}I}{{\partial y}^{2}} \end{matrix} \right)$ (1)

where $x$ and $y$ are the Cartesian axes. If the eigenvalues of the Hessian matrix were set as $|\lambda_{1}|\approx0$, $|\lambda_{1}|<<{|\lambda}_{2}|$, the Hessian-based vascular filter is given by Equation 2,

$V\left( \sigma\right)=\left\{ \begin{aligned} 0 \mathrm{if} \lambda_{2}>0, \\ {(exp(-\frac{\mathcal{R}_{B}^{2}}{2\beta^{2}})}^{2}(1-{\exp( -\frac{S^{2}}{2c^{2}})}^{2}) \mathrm{else}. \end{aligned} \right.$(2)

where $\mathcal{R}_{B}=\frac{\lambda_{1}}{\lambda_{2}}$ is a blobness measure, $S=\sqrt{\lambda_{1}^{2}+\lambda_{2}^{2}}$ is a background measure, $\sigma=r\sqrt{2}$ specifies the vessel diameter, $r$ is the vessel radius, and $\beta$and $c$ are thresholds controlling the sensitivity of the filter in being able to the measure $\mathcal{R}_{B}$ and $S$. In our study, $\beta$ and $c$ were fixed at 0.01 and 0.04, respectively, and the $\sigma$ values ranged from 0.5 to 2.5 with an increment of 0.05.

**Appendix E4**

**Peritumoral vessel segmentation by algorithm for algorithm segmentation.** Since the intensity range of MR images acquired from the different scanners were different, the image normalization process was used to normalize the gray values of MR images. In this process, the image was normalized by centering it at the mean with standard deviation. The gray values of MIP image were normalized by using the standardization method with a scale of 100. The equation of the image normalization was as follows:

$$I_{Normalize}=\frac{I-\mu_{I}}{\sigma_{I}}\times100$$

Where $I$ denotes the original intensity of MIP image, $I_{Normalize}$ is the normalized intensity of MR image, $\mu_{I}$ is the mean value of the image intensity, and $\sigma_{I}$ is the standard deviation of the image intensity.

For peritumoral vessel segmentation, anatomic breast segmentation was performed by delineating the breast-chest wall and breast-air margins using the region-growing algorithm, where the initial seeds were selected, and the neighboring pixels were automatically added if the pixel matched the seed ROI intensities. The gray integration projection algorithm was applied to determine the breast boundaries and separated the left and right breasts on MIP images. The lateral side of the breast tumor was identified according to the tumor location.

The enhancement and segmentation of peritumoral vessels were performed by the eigenvalue analysis of the multiscale Hessian-based filter. After enhancing vessel structures in the MIP images, a histogram-based threshold algorithm was used to segment the peritumor vasculature from the background. A fixed-size region (50 mm × 100 mm) was applied to the mammary vessel region, and 99% of the ROI histogram was selected as the threshold to extract vessel-like structures. To reduce small gaps and fill holes in the resulting binary mask, median filter and morphology operators were applied to remove small isolated objects and connect broken vessels. The binary vessel segmentation region and the lateral tumor breast mask were intersected to obtain the peritumoral vasculature by algorithm. The senior breast radiologist (xx, 11 years of experience) performed manual editing based on the vasculature segmented by algorithm to get the final peritumoral vasculature

**Appendix E5**

**The correct-detection rate, incorrect-detection rate, and missed-detection rate for algorithm segmentation.** The final peritumoral vasculature, checked and edited by a breast radiologist with 11 years of experience, represented the reference standard. The radiologist compared the final vasculature images and vasculature images segmented by algorithm, and identified the number of vessels correctly detected, the number incorrectly detected, and the number undetected. The correct-detection rate was defined as the number of vessels correctly detected by the algorithm over the number of vessels checked by the radiologist. The incorrect-detection rate was defined as the number of non-vessels checked by the radiologist over the number of vessels detected by the algorithm. The missed-detection rate was defined as the number of non-vessels detected by the algorithm over the number of vessels checked by the radiologist.

**Appendix E6**

**Comparison of model performance for three datasets by evaluating on metrics of accuracy, sensitivity, specificity, PPV, NPV, precision and recall, respectively.**

| **Model** | **Dataset** | **Accuracy** | **Sensitivity** | **Specificity** | **PPV** | **NPV** | **Precision** | **Recall** |
| --- | --- | --- | --- | --- | --- | --- | --- | --- |
| Vessel | Primary cohort | 73.45 | 70.45 | 75.36 | 64.58 | 80.00 | 64.58 | 70.45 |
|  | Internal validation cohort | 58.06 | 82.35 | 44.07 | 45.90 | 81.25 | 45.90 | 82.35 |
|  | External validation cohort | 56.60 | 81.25 | 45.95 | 39.39 | 85.00 | 39.39 | 81.25 |
| Tumor | Primary cohort | 64.60 | 65.91 | 63.77 | 53.70 | 74.58 | 53.70 | 65.91 |
|  | Internal validation cohort | 54.84 | 64.71 | 49.15 | 42.31 | 70.73 | 42.31 | 64.71 |
|  | External validation cohort | 60.38 | 62.50 | 59.46 | 40.00 | 78.57 | 40.00 | 62.50 |
| Fusion | Primary cohort | 75.22 | 70.45 | 78.26 | 67.39 | 80.60 | 67.39 | 70.45 |
|  | Internal validation cohort | 61.29 | 70.59 | 55.93 | 48.00 | 76.74 | 48.00 | 70.59 |
|  | External validation cohort | 62.26 | 68.75 | 59.46 | 42.31 | 81.48 | 42.31 | 68.75 |

*PPV*; *NPV* negative prediction value.
